# Submental versus platysma flap for the reconstruction of complex facial defects following resection of head and neck tumors

**DOI:** 10.12669/pjms.304.5177

**Published:** 2014

**Authors:** Jawad Safdar, Fa-Yu Liu, Yousuf Moosa, Zhong-fei Xu, Zhen-ning Li, Chang-Fu Sun

**Affiliations:** 1 Jawad Safdar, MD, Department of Oromaxillofacial-Head and Neck Surgery, School of Stomatology, China Medical University, No. 117, Nanjing North Street, Heping District, Shenyang, Liaoning 110002, PR China.; 2 Fa-Yu Liu, MD, Department of Oromaxillofacial-Head and Neck Surgery, School of Stomatology, China Medical University, No. 117, Nanjing North Street, Heping District, Shenyang, Liaoning 110002, PR China.; 3 Yousuf Moosa, MD, Department of Periodontology, School of Stomatology, China Medical University, No. 117, Nanjing North Street, Heping District, Shenyang, Liaoning 110002, PR China.; 4 Zhong-fei Xu, Department of Oromaxillofacial-Head and Neck Surgery, School of Stomatology, China Medical University, No. 117, Nanjing North Street, Heping District, Shenyang, Liaoning 110002, PR China.; 5 Zhen-ning Li, MS, Department of Oromaxillofacial-Head and Neck Surgery, School of Stomatology, China Medical University, No. 117, Nanjing North Street, Heping District, Shenyang, Liaoning 110002, PR China.; 6 Chang-Fu Sun, MD, Department of Oromaxillofacial-Head and Neck Surgery, School of Stomatology, China Medical University, No. 117, Nanjing North Street, Heping District, Shenyang, Liaoning 110002, PR China.

**Keywords:** Oncology, Platysma flap, Reconstructive surgery, Submental flap

## Abstract

***Objective:*** To compare the platysma flap with submental flap in terms of tumor and flap characteristics, operative properties and the functional outcomes.

***Methods:*** A total of 65 patients presented with tumors of head and neck and underwent curative tumor resection with different neck dissections at the Department of Oromaxillofacial-Head and Neck Surgery, School of Stomatology of China Medical University; from March 2005 to December 2012 were included in the study. After radical tumor excision and neck dissection the resultant complex defects were reconstructed with either platysma flap or the submental flap. The extent of surgical resection, the type of neck dissection and choice of flap reconstruction was at the discretion of the surgical team. The functional outcomes, operative time and characteristics of both platysma and submental flaps were compared and the statistical tests of significance were applied accordingly.

***Results:*** The mean age was 60 years. The complex facial defects of 30 patients were reconstructed with platysma flap and of 35 patients with submental flap. Mean operation time of submental flap including flap harvesting (5.58±1.96hrs) was shorter than platysma flap (6.2±1.4hrs). The majority of the flaps (88-93%) were taken successfully in both groups. Submental flap was associated with significantly higher patients’ satisfaction regarding acceptable functional outcomes (p-value 0.027). The mean reduction in mouth opening was significantly smaller in platysma group (0.37 ±0.18cms) than the submental group (0.47±0.16).

***Conclusion:*** This study demonstrates that both platysma and submental flap techniques can be used for the reconstruction of complex facial defects with the acceptable functional outcome. The platysma flap can be harvested to medium size defects up to 70cm^2^ with good mouth opening. The submental flap is simpler, faster with a wider range of application and more acceptable functional outcomes.

## INTRODUCTION

Head and neck tumors are the sixth most common cancer in the world.^1^ The operable cancers are treated with curable resections with radical or selective neck dissection. These radical surgeries results in complex myocutaneous facial, oral and neck defects with severe functional impairment in most of the cases. Their reconstruction with good cosmesis, anatomic integrity and early restoration of mouth function and swallowing is always a challenging task for the surgeons.^2^ Various reconstruction methods such as Mucosal defects, skin grafting, local flaps, pedicled myocutaneous flaps, free flaps can be performed depending on the size and location of the defects. Generally smaller defects heal by secondary intention while for the larger defects the radial forearm flap, anterolateral thigh flaps have been used for 20 years.^2^^,^^3^ With these techniques are preferred variably in different institutions depending upon the availability of resources, expertise available and the type and the extent of resections performed.^4^

The pedicled myocutaneous flaps are useful alternative for the reconstruction of complex facial defects if micro vascular free tissue transfer facilities and expertise are not available.^5^ Studies show that early restoration of facial anatomy and function with minimal limitation in mouth opening can be achieved with these flaps.^6^^,^^7^ An ideal pedicled flap should be thin, pliable, with a long vascular pedicle and good facial color match.^8^ The myocutaneous platysma and submental flaps are commonly used for the reconstructions of small to medium sized facial, oral and neck defects.^9^^-^^11^ The submental flap is thin skin, long vascular pedicle, close proximity to facial and intraoral defects in the well hidden donor site concealed under horizontal ramus of the mandible, make the submental flap preferable. But this flap can also be associated with some limitations such as risk of nodal metastasis, difficulty in clearing the level I lymph nodes and in addition to its contraindications which include previous radiotherapy, ligation of the facial artery or prior neck surgery.^10^^,^^11^ On the other hand flexibility, good color and texture match are the features of platysma flap for reconstruction along with good functional outcomes due to capability of closing medium sized defects associated with head and neck can be pedicled either superiorly, inferiorly or posteriorly. But it is also the fact that the defect of bigger sizes can lead to greater scar with long surgical time and compromised surgical outcomes. Insufficient mass, partial dependence on the facial artery and venous congestion of the platysma flap lead to its contra indication in cases where the patient presents with bulky nodal disease with a need to sacrifice vessels or external jugular vein.^9^^,^^12^^-^^14^

There are number of recent studies, preferring one of the two flaps, but no study has been published so far comparing the two commonly performed techniques. Therefore, this study aims to compare the platysma flap with submental flap in terms of tumor and flap characteristics, operative properties and the functional outcomes.

## METHODS

Total 65 patients were included in this study from March 2005 to December 2012. These patients received surgical reconstruction of Platysma flap and Submental flap at the department of Oromaxillofacial Head and Neck surgery, School of Stomatology of China Medical University. Patients with widespread metastatic condition and inoperable cancers were excluded. The protocol of our study was approved by the institutional review board and conducted in accordance with the declaration of Helsinki. Written Informed consent was obtained from all the patients.

**Fig.1:**
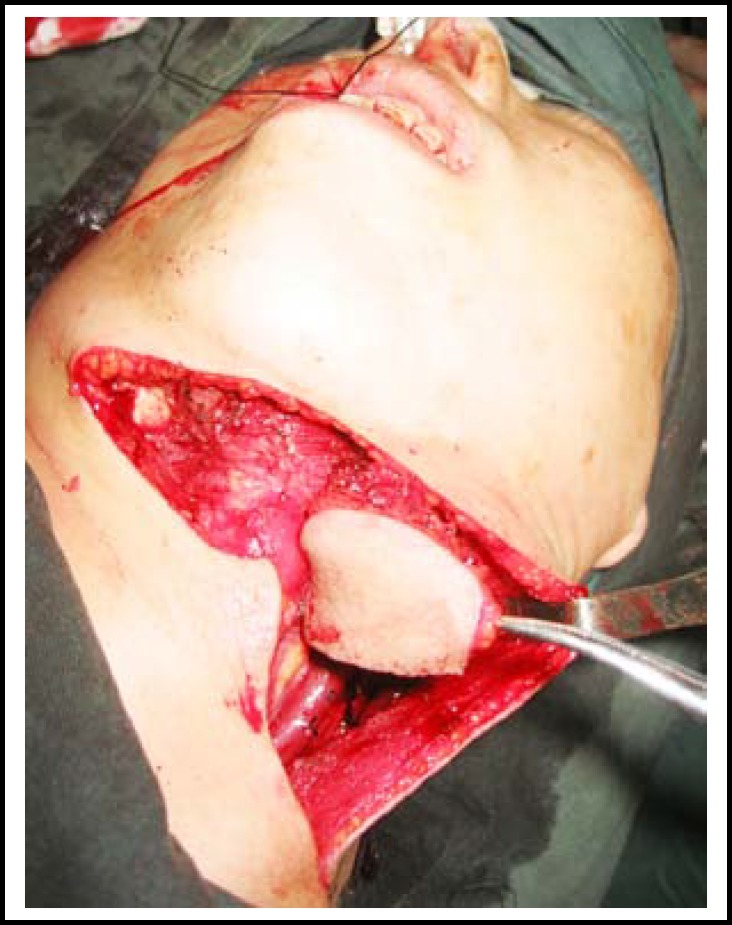
Submental flap is raised

**Fig.2:**
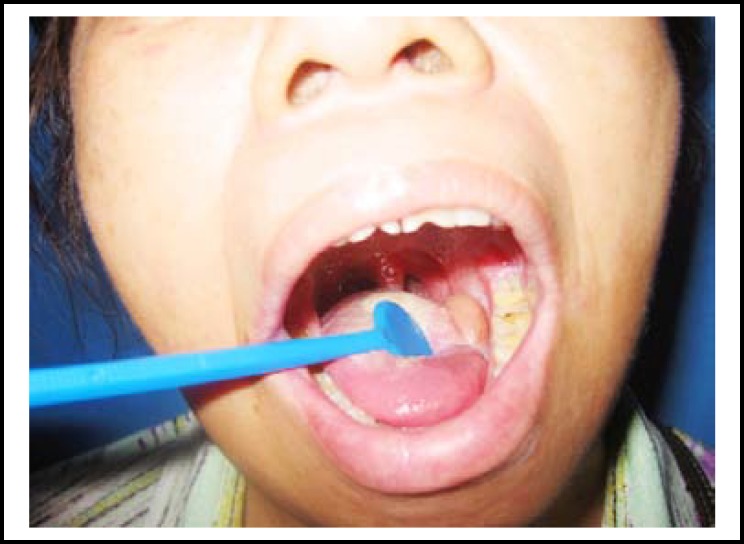
Postoperative result of submental flap after one year

**Fig.3:**
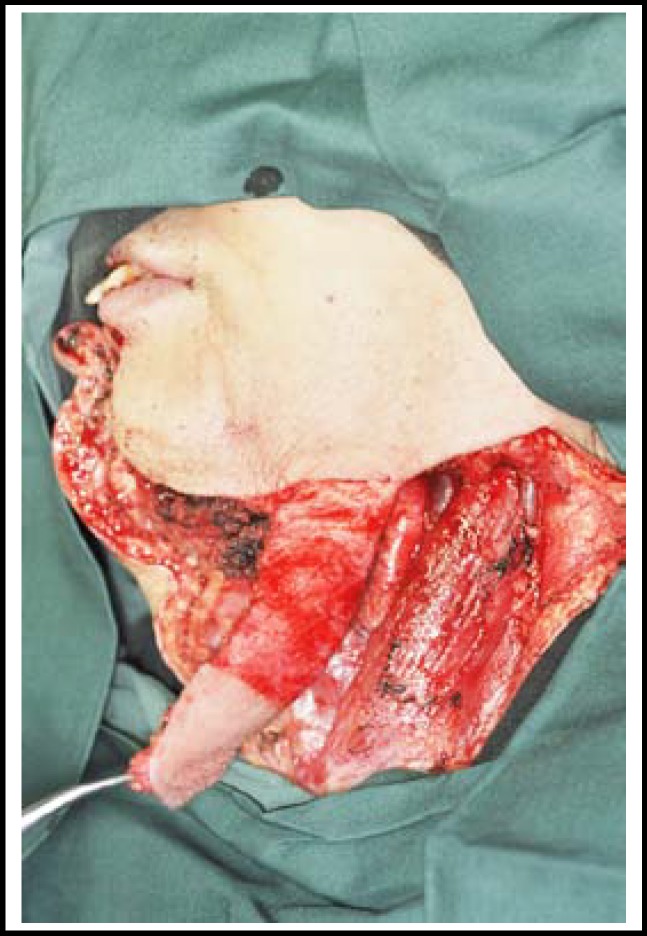
Platysma flap is raised

**Fig.4:**
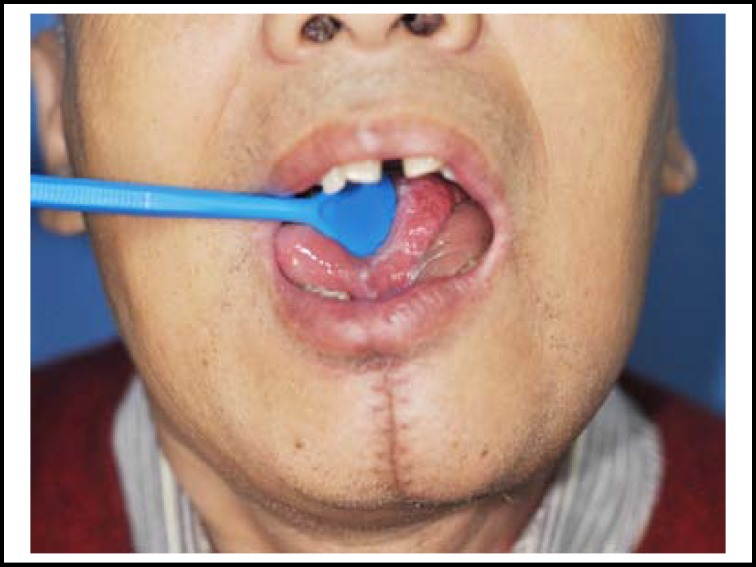
Postoperative result of platysma flap after 8 months

In this study, we divided the patients into PT and SM groups. PT represents patients who had received Platysma flap and patients with Submental flap were included in SM group. After radical tumor excision and neck dissection the resultant complex defects were reconstructed with either platysma or submental flaps (Fig. 1-4). All the surgeries were performed by experienced, well skilled surgeons in both flap techniques. The extent of surgical resection, the type of neck dissection and choice of flap reconstruction was at the discretion of the surgical team. The Harvesting technique for both flaps was performed as described by our colleagues.^5^^,^^15^ Histologically, we confirmed tumor free margin of resection by using a frozen section technique. The size of the flap was designed according to the anticipated defect resulting from the excision of the primary tumor and neck dissection.

A detailed performa was used to document patients’ demography, Comorbidities, tumor site, stage, operative time, surgery and neck dissection performed. Functional outcome was assessed in terms of flap viability, flap complications, post-operative reduction in mouth opening, hospital stay and tumor recurrence. Postoperative function such as speech and swallowing was measured according to the method mentioned by Peng et al.^14^ and Hell et al.^16^ which states that a score of 7 represents an excellent, while 6 and 5 scores as better and less than 4 as poor. A score of not less than 5 is taken as satisfactory result. The width of mouth opening was obtained by measuring the distance between the incisal edges of upper and lower central incisors before surgery and 6 months later to surgery.

The functional outcomes, operative time and flap characteristics of the two groups were compared and the statistical tests of significance were applied accordingly. Chi square test was applied to compare the frequency and percentages of categorical variables. T-test was used to compare means of numerical variables and enumerate level of significance. The difference in average age and mean operation time between the two flap techniques was evaluated by Mann –Whitney test. The level of significance was set at p-value < 0.05.

## RESULTS

A total of sixty five patients with tumors of head and neck were included in the study. The mean age was 60 years, 45 patients were male and 20 were females with male to female ratio of 2.25:1. The complex facial defects of 30 patients were reconstructed with platysma flap and 35 patients with submental flap. Most common histologic diagnosis was squamous cell carcinoma (80%) and frequently involved surgical defect site was the tongue in both groups. Demographic profile with Comorbidities of patients is shown in (Table-I).

All patients underwent curative tumor resections with different neck dissections. Twenty four patients had tumors of Tongue (36.9%), 10 patients with tumor on Floor of the mouth (15%), 6 patients with tumor on Buccal Mucosa (9.2%), 4 patients with tumor on Lip (6%), 4 gingival tumor patients (6%), 3 patients with tumor on face (4.6%) and others 14 patients with tumors on different parts of head and neck including parapharyngeal space, parotid region (21.5%) and were reconstructed with both flap techniques. Tumors of alveolar ridge of 2 patients (3%) and 3 patients of sublingual gland tumor (4.6%) were reconstructed specifically with submental flap. Most (38/65) of the patients (58.4%) had advance tumor i.e. T_3_ and T_4_ and (27/65) of the patients (41.5%) had T_1 _and T_2_. Primary closure was used for all donor sites. The tumor characteristics and type of neck dissections are shown in (Table-II.) The mean platysma flap size was 39.35±13.15cm and submental flap was 27.99±12.57cm. The majority of the flaps (88-93%) were taken successfully in both groups. In platysma flap group single patient developed infection, while other patients developed hematoma and partial flap necrosis. In submental flap group necrosis of the distal flap tip was observed in two patients, hematoma and infection were also seen in one patient. Wound dehiscence was also observed in two patients of PT group and in one patient of SM groups. No patients developed a total flap loss. Hematoma was drained, the infection treated with antibiotics and distal tip necrosis recovered gradually. During follow up, recurrence was observed in 7 (20%) patients in submental flap group as compare to 11 (36.7%) patients in platysma flap group; however time to recurrence was longer (14 months) in platysma flap group patients than submental flap group patients (11 months).

There was significant difference regarding reduction in mouth opening (p-value 0.018) between two groups. Most (12) of the patients were unsatisfied because of limited tongue movement in varying degrees, especially after resection and reconstruction of tumors of the tongue. Total 3 patients in platysma flap group and 7 out of 9 in submental flap group had limited movement of the tongue. In remaining two patients, one patient with tumor of parapharyngeal space and one patient with tumor on floor of the mouth had limited tongue movement in submental flap group. Three patients in platysma flap group and one patient in submental flap group had a speech problem respectively. Neck stiffness were seen in two patients of the platysma flap group and none in submental flap group. Three patients complained of hair growth in the oral cavity in submental flap group and none in platysma flap group (Table-III). Flap characteristics, operative time and functional outcome variables of both groups were compared which are shown in Table-IV. It shows significant differences in both flap techniques in terms of flap characteristics and functional outcome variables, i.e. mean hospital stay (p-value 0.004) and acceptable function (p-value 0.027). Four patients in each group underwent bilateral neck dissections, the mean operative time, including flap harvesting of bilateral neck dissection is higher in both groups. No significant difference was observed in average age (p = 0.757) and average operation time (p = 0.053) between two flaps evaluated by Mann-Whitney test.

## DISCUSSION

Selection of different flap technique depends on various factors such as patient status, functional result, flap reliability. In this retrospective study, we observed several significant differences in the two commonly performed flap techniques. Platysma flap (39.35±13.15cm) was harvested successfully to larger surface area as compared to submental flap (27.99±12.57cm). However, it was associated with longer operative time and hospital stay (8.73±1.72) and a higher percentage of impaired function. Submental flap showed a wider range of usage due to its proximity to oral cavity and was more acceptable to the patients with lesser difficulty and limitation in tongue and neck movements.

**Table-I:** Patients demography& comorbidities

	***Platysma flap***	***Submental flap***
Age (mean±SD)	59.70±11.49	60.60±13.42
Male/female ratio	21/9	24/11
Co Morbidity	14(46.7)	18(51.4)
Hypertension	13(43.3)	10(28.57)
Diabetes	4(13.3)	3(8.57)

**Table-II:** Tumor characteristics & neck dissection

		***Platysma flap***	***Submental flap***	***Total***
Tumor location	Tongue	13	11	24
	Buccal mucosa	3	3	6
	Face	1	2	3
	Gingival	3	1	4
	Floor of mouth	6	4	10
	Alveolar ridge	0	2	2
	Lip	1	3	4
	Parotid region	1	1	2
	Sublingual gland	0	3	3
	Para pharyngeal space	1	2	3
	Tongue +Para pharyngeal	1	1	2
	Tongue+ floor of mouth	0	2	2
Tumor T-stage	T1	2	3	5
	T2	11	11	22
	T3	3	11	14
	T4	14	10	24
Neck dissection	Radical Neck Dissection	11	0	11
	Mod Radical Neck Dissection	2	6	8
	Selective Neck Dissection	17	29	46

**Table-III:** Comparison of Complications of Platysma flap and Submental flap

	***Platysma flap***	***Submental flap***	***(p value)***
Mean reduction in mouth opening	0.37+0.18	0.47+0.16	0.018*
Limited tongue movement	3	9	0.10
Neck stiffness	2	0	0.12
Speech problems	3	1	0.23
Infection	1	1	0.91
Necrosis of distal flap tip	0	2	0.18
Partial flap necrosis	1	0	0.27
Hematoma	1	1	0.91
Hair growth	0	3	0.10
Wound Dehiscence	2	1	0.46

Chi^2^ test was applied between groups with fisher exact test applied in small data.

* student t test applied between the groups. Significant p-value<0.05

**Table-IV:** Comparison of flap characteristics & functional outcome

	***Platysma flap***	***Submental flap***	***(p value)***
Mean Flap size	39.35±13.15cm	27.99±12.57cm	<0.01*
Flap Survival	28(93.3%)	31(88.6%)	0.580
Mean operation time (MOT)	6.20±1.47hrs	5.58±1.96hrs	0.156
MOT with unilateral neck dissection	6.01±1.3hrs	5.81±1.9hrs	0.675
MOT with Bilateral neck dissection	7.74±1.5hrs	6.12±2hrs	0.253
Mean hospital stay (days)	8.73±1.72	7.62±1.28	0.004*
Acceptable function%	19(63.3%)	26(74.3%)	0.027*

* significant p-value<0.05

The platysma flap was first introduced by Futrell et al. in 1978 and then widely used for the reconstruction of complex facial defects.^17^ The functional results of platysma flap are comparable to other studies.^9^ Li and colleagues show that partial necrosis of the flap were observed in four (7.4%) patients with flap survival in all patients. However the functional satisfaction was higher (87%) with shorter operation time (5.7±1.17 hours).^15^ Whereas our study showed one patient with Platysma flap reconstruction had developed partial flap necrosis (3.3%) and none of the patient had complete flap necrosis or necrosis of the distal flap tip. (MOT) mean operation time (6.20+1.47 hours), MOT with unilateral neck dissection (6.01+1.3 hours) and MOT with bilateral neck dissection (7.74+1.5 hours) with 63.3% of acceptable function. Fang show that the dissection of platysma was easier than radial forearm free flap but with a less acceptable functional outcomes similar to our study.^3^ Partial necrosis of platysma flap is mainly attributed to venous congestion of the flap which was successfully managed conservatively in most of the studies.^12^^,^^13^ The other study showed 88.5% of platysma flap survival rate,^18^ whereas in our study the survival rate of platysma flap is 93.3%. For better survival of the platysma flap, the flap pedicle should be wide and broadly tunneled, excessive stretching and tight suturing should be avoided and smooth postoperative drainage of blood and seroma should be ensured.^14^

The submental flap showed lower complication rate in terms of functional outcomes and recurrence. Our study showed none of the patient with submental flap reconstruction had developed partial flap necrosis but two patients had necrosis of distal flap tip. Acceptable function was observed (74.3%) in twenty six patients. Mean operation time (5.58+1.96 hours), MOT with unilateral neck dissection (5.81+1.9 hours) and MOT with bilateral neck dissection (6.12+2 hours). Since its first description by Martin et al.^19^ in 1993 it has been widely used by various surgeons worldwide owing to its relative simplicity in raising the flap and lower post-operative morbidity.^10^^,^^11^^,^^20^^,^^21^ The disadvantages are the hair bearing nature in males especially when it was used to reconstruct the intra oral defects and this study also showed hair growth in oral cavities of three patients. Secondly, if the cancer is suspected to involve the submental lymph nodes than the lymph node clearance is jeopardized. Chow addressed these oncologic concerns by reviewing 10 cases of submental artery flap reconstruction after resection of aggressive oropharyngeal cancers. Three cancer recurrences were noted that were related to the aggressive nature of the tumors rather than the ineffective lymphnode clearance.^22^ They recommend that dissection in the proper anatomical (subplatysmal) plane to raise the submental would minimize the chances of tumor spread and inadequate clearance. Amin and colleagues adopted the policy of complete lymph node dissection before flap harvesting and recommend that indiscriminate use of submental flap should be avoided especially in patients with clinically advance nodal disease.^23^ Similarly, in our study 11 patients with advance nodal disease underwent radical neck dissection and all these patients were reconstructed with platysma flap. The concern regarding oncological safety of submental flap was also raised by other authors worldwide.^24^^,^^25^

To the best of our knowledge this is the first study comparing the flap characteristics and functional outcomes of the two commonly performed flap techniques and shows significant differences between the two flaps. However due to technical difficulties and contraindications of either flaps in specific circumstances randomization was not possible. This could be the reason of lack of clinical and randomized controlled trials comparing the two techniques. During the seven year study period, the five year follow up for tumor free survival was not possible in all patients. We also recommend future studies with randomization among patients in selected cases where both the flap techniques can be used safely with longer follow up.

## CONCLUSION

Both platysma and submental flap techniques can be used for the reconstruction of complex facial defects with the acceptable functional outcomes. The platysma flap can be harvested to medium size defects up to 70cm^2^. The submental flap is simpler, faster with a wide arc rotation can be used in different ranges of head and neck defects with better functional outcomes. However, it should be used cautiously in patients with advanced nodal diseases.

## Notes

***Conflicts of interest and Source of funding:*** None declared.
